# Mechanisms of microbe-immune system dialogue within the skin

**DOI:** 10.1038/s41435-021-00133-9

**Published:** 2021-05-15

**Authors:** Nonhlanhla Lunjani, Sinead Ahearn-Ford, Felix S. Dube, Carol Hlela, Liam O’Mahony

**Affiliations:** 1 Department of Dermatology, University of Cape Town, Cape Town, South Africa; 2 APC Microbiome Ireland, University College Cork, Cork, Ireland; 3 Department of Molecular and Cell Biology, Faculty of Science, University of Cape Town, Cape Town, South Africa; 4 Institute of Infectious Disease & Molecular Medicine, University of Cape Town, Cape Town, South Africa; 5 Department of Medicine, University College Cork, Cork, Ireland; 6 School of Microbiology, University College Cork, Cork, Ireland

**Keywords:** Mucosal immunology, Immunological disorders

## Abstract

The prevalence and severity of dermatological conditions such as atopic dermatitis have increased dramatically during recent decades. Many of the factors associated with an altered risk of developing inflammatory skin disorders have also been shown to alter the composition and diversity of non-pathogenic microbial communities that inhabit the human host. While the most densely microbial populated organ is the gut, culture and non-culture-based technologies have revealed a dynamic community of bacteria, fungi, viruses and mites that exist on healthy human skin, which change during disease. In this review, we highlight some of the recent findings on the mechanisms through which microbes interact with each other on the skin and the signalling systems that mediate communication between the immune system and skin-associated microbes. In addition, we summarize the ongoing clinical studies that are targeting the microbiome in patients with skin disorders. While significant efforts are still required to decipher the mechanisms underpinning host-microbe communication relevant to skin health, it is likely that disease-related microbial communities, or Dermatypes, will help identify personalized treatments and appropriate microbial reconstitution strategies.

## Introduction

**Fig. 1:**
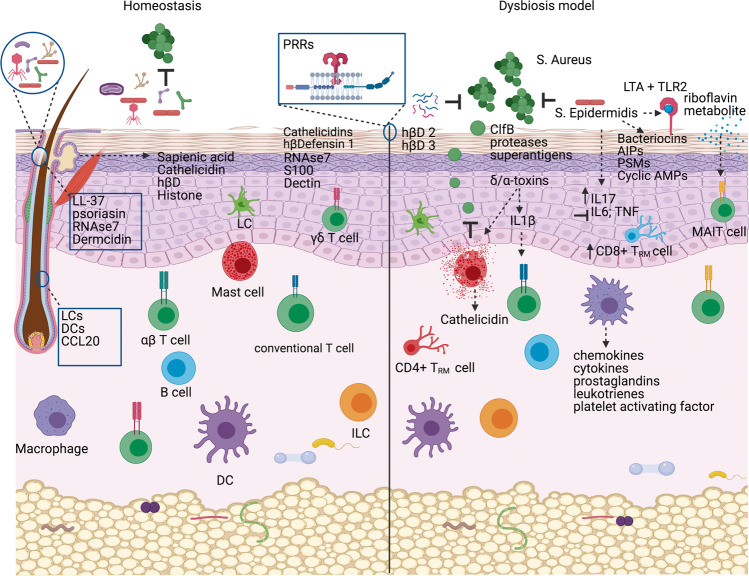
Microbe–microbe and microbe–host interactions on the skin. Diverse microbes on the skin surface and hair follicles interact with each other such that they limit the proliferation of pathogenic organisms. Microbes influence the growth of other microbes via secretion of bacteriocins, auto-induced peptides (AIPs), phenol soluble modulins (PSMs) and cyclic anti-microbial peptides (AMPs). Keratinocytes inhibit microbial growth by constitutively secreting antimicrobial peptides such as cathelicidin and human beta defensins (hβDs). Pattern recognition receptors (PRRs) recognize microbial structures to induce appropriate innate immune responses. Lipotechoic acid (LTA) from Staphylococcus (S.) epidermidis is recognized via toll-like receptor 2 (TLR-2). Mucosa-associated invariant T (MAIT) cells specifically respond to microbial-derived riboflavin metabolites. Innate cells such as Langerhans cells (LCs) and dendritic cells (DCs) sample microbial antigens within the hair follicle, while secretion of chemokines including chemokine (C-C motif) ligand 20 (CCL20) control the recruitment of lymphocyte subsets. Dysbiosis is associated with overgrowth of microbes such as *S. aureus*, which employs clumping factor B (ClfB), toxins, proteases and superantigens to colonize the skin and induce damaging inflammatory responses. Figure created with BioRender.com.

Recent decades have seen a rapid increase in chronic inflammatory disorders due to inappropriate or misdirected immune responses accompanied by insufficient development of immune regulatory networks. It is generally accepted that changes in environment, lifestyle and dietary factors may play a role in the miseducation or deficient training of the immune system [1, 2]. In particular, factors that negatively impact microbial diversity and metabolism are thought to dramatically influence mechanisms of immunological tolerance [3]. An enormous variety of microbes colonize body surfaces and these microbes are organized within complex community structures, utilizing nutrients from other microbes, host secretions and the diet. Modern lifestyles, medications and social interactions have fundamentally altered and disrupted the human microbiome metacommunity and, as a consequence, risk of immune-mediated diseases [4]. The mechanisms that contribute to the intimate and sophisticated inter-kingdom dialogue that maintains a stable environment with important beneficial physiological, metabolic, and immunological effects on the host are being intensely investigated by many research groups across the world. Although exposed to modification by the external environment, human skin actively regulates microbial colonization and microbial entry into dermis/subcutis. Microbes interact with each other and with host cells, including keratinocytes and immune cells, in turn influencing skin homoeostasis (Fig. 1). Some of the host immune functions that are influenced by the skin microbiome include promotion of host defence networks against pathogens, control of inflammation, and education of adaptive immune pathways [5, 6]. Commensal skin microbiota can directly inhibit colonization and invasion by pathogenic microbes or opportunistic microbes. For example, *Staphylococcus epidermidis* (*S. epidermidis* is one of the most abundant commensal species in the skin) can stimulate keratinocyte antimicrobial peptides production, as well as inhibit inflammatory cytokine release and inflammation during wound healing [7]. In this review, we will summarize some of the key recent findings that identify novel mechanisms relevant to the unique features of host–microbe interactions in the skin and discuss the potential for novel preventative and therapeutic approaches using microbes or microbial metabolites for skin health.

## The importance of the skin habitat

The composition and function of human skin is such that it does not only form a barrier to the external environment but serves as dynamic ecosystem consisting of living and non-living components that dictate the local environmental and nutrient conditions of the skin surface. Components of this ecosystem are highly interactive and function together as a sophisticated system. Different methodologies (culture and non-culture based) have provided precision and resolution in surveying skin microbial communities, confirming the presence of the bacterial, fungal, viral and mite communities in the skin. The emerging picture indicates that the skin harbours a diverse population of microbes whose composition is largely determined by site-specific physiological factors, such as moisture and sebum content [8, 9].

At the forefront is the highly keratinized epidermis, the result of a specialized differentiation process of keratinocytes (the main cell type in the epidermal barrier). The uppermost layer of the epidermis, the stratum corneum, harbours a rich diversity of microbes, contributing to the barrier properties of the skin [10]. Current detection techniques have shown that microbes reside not only on the external interfollicular epithelial surface but also on the entire skin appendage surface and even below the basement membrane, extending to the dermis and dermal adipose tissue [11]. Appendage structures including sebaceous glands, hair follicles, eccrine ducts, and apocrine ducts maximize epithelial surfaces for microbial attachment and colonization, suggesting that the epithelial surfaces of skin appendages are relevant interfaces for cross-talk between microbes and the host [12].

Sebum, a lipid-rich substance secreted by the sebaceous glands lubricates the hair and skin. The hydrolysis of sebum by commensal microbes generates free fatty acids such as sapienic acid, which work to control microbial colonization along with sebocyte-derived anti-microbial peptides (AMPs) such as cathelicidin, β-defensins and antimicrobial histones [13]. Eccrine sweat (water, salt and electrolytes) secreted directly onto the skin surface, contributes to the acid mantle of the skin, creating an environment that limits the composition of microbes that can survive and proliferate. The density of eccrine sweat glands impacts the microbial colonization of the skin [14]. *Cutibacterium acnes* is lipophilic and found in abundance in sebaceous skin sites. *C. acnes* is also responsible for acne vulgaris, producing various adhesions, toxins and inflammatory mediators. *Staphylococcal* species are found in moist skin niches, and are halotolerant organisms that have evolved to use urea found in sweat as a nitrogen source. Certain *Staphylococcus* species, such as pathogenic *S. aureus* strains, employ multiple mechanisms in order to colonize the skin. They are able to (i) produce adhesins such as clumping factor B that promote bacterial adherence to corneocytes, (ii) disrupt the epidermal barrier due to α-toxin and various extracellular proteases and (iii) activate inflammatory responses by staphylococcal superantigens [15, 16].

Human skin has approximately 2 million hair follicles. This dermal appendage is home to a unique and complex microbiota including bacteria, fungi, viruses (including bacteriophage) and mites. The follicles form tissue columns within human skin that directly link the skin environment and its surface microbiome with all cutaneous layers, thereby making the skin the largest epithelial surface in the human body for efficient microbe–host immune interactions and an ideal habitat that favours microbial survival. This is kept in check by an inhibitory environment in the hair follicle whereby bacterial metabolites induce AMPs such as cathelicidin LL-37, psoriasin, RNAse 7 and dermcidin. Within the hair follicle, both Langerhans cells and CD11b+ type 2 conventional dendritic cells (cDC2s) acquire and present topical antigen to T cells, and the IRF-4-dependent cDC2s are required for T cell priming and LAP+ regulatory T cell (Treg) expansion [17]. Young mice have reduced Treg cells in the absence of hair follicles. The niche provided by hair follicles accommodates coagulase-negative *Staphylococci* (CoNS) species such that these microbes appear to stimulate hair follicle production of chemokine (C-C motif) ligand 20 (CCL20), which is chemotactic for Treg migration into skin [18]. These resident microbes appear to be necessary for differentiation of skin stem cells and establishment of immune tolerance to commensal microbes. The outer root sheath keratinocytes are able to mobilize inflammatory cells in the event of microbial dysbiosis [19].

The dermis was previously thought to be devoid of a microbial community in the absence of barrier defects. However, this concept has been challenged. DNA from Proteobacteria, including *Burkholderiales* and *Pseudomonadales*, as well as Actinobacteria, have been detected in the subepidermal compartments [20]. Notably, dermal bacterial sequences were dissimilar to those detected on the skin surface and there was no evidence to support the translocation of bacteria via phagocytic cells into the subdermal compartment. The viability of the dermal-associated microbes is yet to be confirmed, but microorganisms need not be alive to exert effects on the host immune system, as discussed in more detail below.

## Microbe–microbe interactions on the skin

Bacterial species colonizing the same ecological niche interact extensively with each other as they compete for nutritional resources. Bacteria may sequester or consume nutrients in a niche preventing their competitors from accessing them, produce antimicrobials that synergise with host-derived AMPs to inhibit the growth of their competitors, or produce factors that interfere with the virulence signalling pathways of their competitors [21–23]. These competitive interactions are important in shaping the composition and diversity of microbial communities and ecosystems, which have important consequences to skin health.

*S. aureus* overabundance, accompanied by a concomitant decline in microbial richness and diversity (especially protective staphylococci) is associated with atopic dermatitis (AD) pathogenesis [24, 25]. Intraspecies competition among *Staphylococci* can inhibit pathogenic strains such as *S. aureus*, by production of (i) bacteriocins, and (ii) autoinducing peptides, which inhibit accessory gene regulator (agr) quorum sensing systems that controls production of virulence factors [26]. The peptides phenol soluble modulin (PSM) γ and PSMδ, produced by *S epidermidis*, limit survival of *S. aureus* on the skin surface. These PSMs cause membrane leakage and membrane perturbation in bacteria, suggesting that these peptides function by a mechanism similar to that of human AMPs. *S epidermidis* strains are also capable of producing specific serine proteases that interfere with *S. aureus* biofilm formation. *Staphylococcus* *hominis* strains can produce *Sh*-lantibiotics α/β- a class of cyclic AMP that contain lanthionine and methyllanthionine. *Staphylococcus* *lugdunensis* also produces the cyclic peptide lugdunin that inhibits *S. aureus* [26–29]. An unidentified antimicrobial factor from *Corynebacterium pseudodiphteriticum* can inhibit *S. aureus* growth and colonization as well as biofilm formation on anterior nares [22, 30].

In addition to antimicrobial factors, signalling inhibitory molecules produced by skin commensal microbes and nasal microbiota can inhibit functioning of the *S. aureus agr* quorum-sensing system in a process called quorum quenching [31–33]. Quorum quenching is associated with reduced *S. aureus* virulence, including inhibition of biofilm formation, haemolytic toxin production and enhanced host immune responses against *S. aureus* in murine skin infection models. In an inflammatory skin disease murine model, treatment with Solonamide B reduced *S. aureus* RNAIII and delta-PSM expression, which was associated with reduced mast cell degranulation and proinflammatory cytokine production [34].

Using *S. aureus* as a model pathogenic microbe, these findings highlight the importance of commensal bacterial species in the prevention of *S. aureus* colonization and pathogenic outcomes during *S. aureus* infections or flare-ups. Skin and nasal commensal microbes produce antimicrobials that limit *S. aureus* growth and colonization, but the relative abundance of these commensals and the potency of their antimicrobial activity against *S. aureus* are often reduced in AD. However, it is too simplistic to suggest that *S. aureus* is the only skin microbe that might negatively influence the skin in patients with AD. Even selected bacterial strains from presumptive commensal species can have detrimental effects. Specific *S. epidermidis* strains can be deleterious to the skin barrier through protease activity that is similar to *S. aureus* [35]. Proteolytic activity was mediated by secretion of an extracellular cysteine protease A (EcpA) controlled by the agr quorum sensing system.

In summary, complex niche-specific ecological networks govern the relative abundance and activity of skin-associated microbes, which have important consequences for skin homoeostasis and health. In addition to *S. aureus*, other potentially damaging microbes such as *C. acnes* are now being shown to be controlled by non-pathogenic microbes [36]. Intervention strategies designed to modify entire communities within a given niche, rather than simply targeting a specific individual microbe, will likely be more effective in the long term.

## Microbe-immune system interactions on the skin

Skin microbiota composition changes during development and is age dependent. In addition, microbial composition is related to changes in host physiology and the influence of external environmental factors. It is also evident that the skin microbiota adapts to prevailing physiological and immunological environment of the sites they inhabit and in turn has unique functional influences on immune maturation and activity in the ecological niches they occupy [37]. While significant skin site specificity is evident, the microbiome usually remains stable at each site over many years.

Neonatal skin is structurally similar to adult skin reaching adult-like maturity at 34 weeks gestation but with very different physiological and immune activity. The skin surface is extensively colonized immediately postpartum with maternally and environment-acquired bacterial strains. This composition is displaced by successful acquisition of further environmental microbes during skin maturation. Early life skin microbial acquisition events and encounters may have long-term health implications through modulation of host immunity and microbe–microbe interactions. In particular, the post-natal period is very important for the development of immune tolerance. Levels of FoxP3+ Treg cells coincide with *S. epidermidis* colonization. Continued exposure to commensal microbes modulates host immune and epithelial cell production of AMPs, cytokines and can inhibit proinflammatory immune activation [38, 39]. Adaptive immune responses in human skin develop during early childhood, however, the neonatal skin is skewed towards anti-inflammatory responses as it’s abundantly populated by Treg cells. Similarly, mice colonized with benign microbial strains in the neonatal period preferentially induce tolerogenic immune responses in skin and gut, while reduced Treg cell numbers have been observed in the skin of young mice raised under germ-free conditions [40, 41].

The early life human skin microbiome stabilizes at about 3 years of age, but then goes through marked changes at the onset of puberty due to hormonal influences on skin physiology, notably sebum production that supports a lipophilic microbiota. Skin physiology in the elderly is altered by several host factors such as hormones and diminished cellular metabolism including immunosenescence. This leads to shifts in microbial composition of the skin. A decline in *Propionibacteria* correlates with a decline in sebum production. In contrast, Archaea relative abundance increases with lower sebum levels. Commensal fungi such as *Malassezzia* seem to remain stable with advancing age.

The beneficial effects on host immune maturation mediated by diverse environmentally acquired microbial exposures have been suggested by several studies. It has been shown that human skin shares numerous common bacterial taxonomies with soil microbes [42]. Recently, environmental biodiversity was deliberately manipulated to examine its effects on commensal skin microbiome and the immune system in young children. The intervention entailed enrichment of urban daycare centre yards for 28 days with segments of forest floor, sod, planters for growing annuals, and peat blocks for climbing and digging. Increased microbial biodiversity was associated with changes in the skin and gut microbiota of children, which, in turn, were related to changes in plasma cytokine levels and regulatory T-cell frequencies [43]. Specifically, the intervention was associated with a shift toward a higher ratio between plasma Interleukin-10 (IL-10) and IL-17A cytokine levels and a positive association between Gammaproteobacterial skin diversity and regulatory T cell frequencies in blood, suggesting that the intervention may have stimulated immunoregulatory pathways. After the trial, children in the intervention daycare centre had more diverse skin Proteobacterial and Gammaproteobacterial communities than children in standard daycare settings. These results demonstrate how environmental biodiversity can promote or prevent the loss of skin bacterial species.

In addition to the epidermis providing a formidable physical barrier and a mutually beneficial habitat for selected microbes, it also supports well-choreographed immune functions. AMP secretion and expression of Pattern Recognition Receptors (PRRs) by keratinocytes regulate microbial density and community composition. Microbes interact with keratinocytes to limit the potential overgrowth of pathobionts. Keratinocytes express several PRRs that are able to distinguish a wide variety of microbial components, including Toll-like receptors (TLRs) bacterial peptidoglycan sensing Nod-like receptors (NLRs), and NLR pyrin domain-containing proteins that sense viral, fungal and self-proteins [44]. Keratinocyte expression of RIG-I-like receptors (RLRs) enable detection of viral RNA, whereas antifungal immunity is tailored by non-TLR signalling such as dectin-1 [45]. RNAse 7 is constitutively released by keratinocytes and has potent antimicrobial activity on a broad spectrum of microorganisms. Damage-associated molecular patterns such as the S100 proteins on keratinocytes can inhibit microbes, with S100A7 also exhibiting a chemotactic function [46]. In addition to the direct microbe–microbe interactions described above, commensal organisms can also augment host defenses. For example, PSMγ and δ from *S. epidermidis*, which have direct antimicrobial effects on *S. aureus*, also activate TLR-2 and enhances tight junction barrier function, induces keratinocyte-derived AMP, induces IL-17 production while inhibiting inflammatory cytokines such as IL-6 and TNF-alpha [47]. Another *S. epidermidis*-derived TLR-2 ligand, lipotechoic acid, inhibits proinflammatory signals following epithelial injury and enhances CD8^+^ skin-resident T cell functions. These *S. epidermidis* specific CD8^+^ T cells express immunoregulatory and tissue repair gene signatures [5]. Tissue resident memory T cells (T_RM_) have been identified in human skin in areas of the previous infection. CD8+ T_RM_ cells locate themselves in the epidermis and CD4+ T_RM_ cells in the dermis, which corresponds with similar T_RM_ migration patterns observed in mouse studies of HSV-1 infection [48]. The extent to which specific commensal microbes influence T_RM_ cells has yet to be determined.

Microbes have direct and indirect effects on the skin barrier. Filaggrin, a key skin barrier protein with an important role in AD pathogenesis, and epidermal lipids influence microbial growth. Overgrowth of certain pathogens stimulate cytokine and chemokine secretion by keratinocytes that then direct respective innate effector cell and adaptive immune cellular function. Damaging the skin barrier can lead to epicutaneous senitization via the TSLP-basophil-IL-4 axis [49]. The skin microbiome negatively or positively influences the skin epithelial barrier [50]. A characteristic microbiome signature has often been described for AD and this pattern was recently shown to associate with the expression of type 2 inflammation pathway genes such as IL-4R, C-C motif chemokine receptor type 4 (CCR4), and C- C motif chemokine 22 (CCL22) in lesional compared with non-lesional skin [51]. Biological processes related to keratinization were the key host response pathways identified in the dry/lipid-poor microenvironments such as the upper leg. *S. aureus* abundance was associated with enrichment for genes important for extracellular matrix organization and leucocyte migration to the skin. In contrast, *S. epidermidis* was associated with enrichment for genes related to epidermis development.

Specialized antigen-presenting cells such as epidermal dendritic cells known as Langerhans cells are located above the basal keratinocyte layer. Their dendrites are able to project towards the horny layer in order to sample microbial components. It has been shown that they efficiently prime immune responses to *C. albicans* and *S. aureus* thereby inducing requisite effector T-cell responses. Langerhans cells are able to sample bacterial toxins, and favour specific humoral immune responses whilst ensuring that the epithelial barrier remains intact. CD1c+ dendritic cells (DCs), CD14+ dendritic cells (DCs of monocyte origin), and CD141+ DCs have a role in antiviral immunity and present antigen for CD8+ T-cell responses. Following pathogen detection, activated skin macrophages rapidly produce chemoattractants, cytokines, prostaglandins, leukotrienes and platelet activating factor [52].

Mast cells (MCs) are located in the upper dermis and contribute to maintaining microbiome-tissue homoeostasis [53]. They are able to produce AMPs such as cathelicidin and have direct bactericidal activity. MCs can recognize microbes through different mechanisms including direct binding of pathogens or their components to TLRs, NLRs, RLRs, and activation of complement receptors. Once microbes activate these receptors, inflammatory mediators are released. These mediators contribute to effective antimicrobial immune responses. In AD, *S. aureus* peptidoglycans recruit MCs and *S. aureus* delta toxins induce MC degranulation, which damage the epithelial barrier and further promote innate and adaptive inflammatory responses [54]. *S. aureus* α-toxins can also induce IL-1β production from skin monocytes, which may further promote effector T cell responses.

Commensal microbes control mucosal-associated invariant T (MAIT) cells. These are an evolutionarily conserved T-cell subset, which represents the most abundant T-cell subset recognizing bacterial compounds [55, 56]. They react to many bacterial species through T-cell receptor (TCR)-mediated recognition of metabolites derived from the vitamin B2 biosynthetic pathway. MAIT cells reside in peripheral tissues during homeostatic conditions with the microbiota seemingly a strong determinant of MAIT cell numbers. The MAIT17 subset, compared to MAIT1 cells, home preferentially to barrier tissues such as the skin, lung and gut. MAIT cells are important for the clearance of bacterial infections [57]. Their role in defence against viral infection has also been noted. In addition to anti-microbial activity, MAIT cells improve wound healing in the skin and are also thought to regulate lung and intestinal epithelial integrity, suggesting a role in epithelium homoeostasis through bi-directional interactions with the local microbiota. In keeping with these observations, blood MAIT cell frequency is modified in inflammatory disease where microbial dysbiosis is a pathogenic feature [58]. As MAIT cell development in mice is restricted to early life, it is possible that dysregulated immune–microbe cross-talk during childhood may negatively affect MAIT cell function throughout life.

## Human clinical trials investigating microbial treatments for skin conditions

Over the past decade, a number of clinical trials evaluating the efficacy of probiotic and prebiotic interventions in dermatologic diseases have been published with mixed results (summarized in Table 1).

**Table 1:** Human clinical trials investigating microbial treatments for skin conditions.

Route of Administration	Condition	Author, year	Study Type	Participants	Intervention	Key findings
Oral	Atopic Dermatitis (AD)	Navarro-Lopez et al., 2018 [59]	RCT	50 children (4–17 years) with AD	*Bifidobacterium lactis* CECT 8145, *Bifidobacterium longum* CECT 7347, and *Lactobacillus casei* CECT 9104 (10^9^ total colony-forming units), daily for 12 weeks (*n* = 26)	Significant reduction in disease severity and topical steroid use in probiotic group Nonsignificant reduction of inflammatory markers (IL-4, IL-5, and IL-13) in probiotic group versus placebo
		Wu et al., 2017 [60]	RCT	66 children (4–48 months) with AD	*Lactobacillus rhamnosus* (MP108) (350 mg), daily for 8 weeks (*n* = 33)	Significant reduction in disease severity in probiotic group versus placebo No difference in topical steroid use between groups
		Woo et al., 2010 [61]	RCT	75 children (2–10 years) with AD	*Lactobacillus sakei* KCTC 10755BP (5 ×10^9^ colony-forming units), twice daily for 12 weeks (*n* = 41)	Significant reduction in disease severity and inflammatory markers (CCL17 and CCL27) in probiotic group versus placebo
		Wang and Wang, 2015 [62]	RCT	220 children (1–18 years) with AD	*Lactobacillus paracasei* (LP) (2 × 10^9^ colony-forming units) or *Lactobacillus fermentum* (LF) (2 × 10^9^ colony-forming units) or LP + LF mixture (4 × 10^9^ colony-forming units), twice daily for 12 weeks (*n* = 55 for each group)	Significant reduction in disease severity in all treatment groups versus placebo
		Prakoeswa et al., 2017 [63]	RCT	22 children (0–14 years) with AD	*Lactobacillus plantarum* IS-10506 (10^10^ colony-forming units/day), twice daily for 12 weeks (*n* = 12)	Significant reduction in disease severity and inflammatory markers (IL-4, IFN-γ, and IL-17) in probiotic group versus placebo
		Han et al., 2012 [64]	RCT	118 Children (1–13 years) with AD	*Lactobacillus plantarum* CJLP133 (0.5 × 10^10^ colony-forming units), twice daily for 12 weeks (*n* = 58)	Significant reduction in disease severity in probiotic group versus placebo and in total eosinophil count as compared to baseline
		Yeşilova et al., 2012 [65]	RCT	40 children (1–13 years) with AD	*Bifidobacterium bifidum*, *Lactobacillus acidophilus*, *Lactobacillus casei*, and *Lactobacillus salivarius* (2 × 10^9^ colony-forming units), daily for 8 weeks (*n* = 20)	Significant reduction in disease severity and inflammatory markers (IL-5, IL-6, IFN-γ, and total serum IgE) in probiotic group versus placebo
		Wickens et al., 2012 [66]	RCT	474 infants at high risk of AD	*Lactobacillus rhamnosus* HN001 (6 × 10^9^ colony-forming units/day) (*n* = 157) or *Bifidobacterium animalis* subsp *lactis* HN019 (9 × 10^9^ colony-forming units/day) (*n* = 158), daily maternal supplementation from 35 weeks gestation until birth, continuing to 6 months in mothers after birth if breastfeeding, and infant supplementation until 2 years	Significant reduction in the cumulative prevalence of AD and nonsignificant reduction in cumulative prevalence of severe disease in *Lactobacillus rhamnosus* HN001 group versus placebo No significant effects on any outcome measures were reported in *Bifidobacterium animalis* subsp *lactis* HN019 group
		Nermes et al., 2011 [67]	RCT	39 infants with AD	*Lactobacillus rhamnosus* GG (ATCC 53103) (3.4 × 10^9^ colony-forming units/day) for 3 months (*n* = 19)	Significant reduction in the proportions of IgA- and IgM-secreting cells in probiotic group Significant increase in the proportions of CD19^+^ CD27^+^ B cells in probiotic group No significant difference in reduction in disease severity between groups
		Rautava et al., 2012 [68]	RCT	205 mother-infant pairs (mothers with allergic disease and atopic senitization)	*Lactobacillus rhamnosus* LPR and *Bifidobacterium longum* BL999 (LPR + BL999) (*n* = 73) or *Lactobacillus paracasei* ST11 and *Bifidobacterium longum* BL999 (n = 70) (1 × 10^9^ colony-forming units), daily maternal supplementation for 2 months before delivery and during first 2 months of breastfeeding	Significant reduction in the risk of developing AD during first 24 months of life in both probiotic groups versus placebo
		Kim et al., 2010 [69]	RCT	112 pregnant women (with a family history of allergic disease)	*Bifidobacterium bifidum* BGN4, *Bifidobacterium lactis* AD011, and *Lactobacillus acidophilus* AD031 (1.6 × 10^9^ colony-forming units of each strain), daily from 8 weeks before delivery up to 6 months after delivery (*n* = 57)	Significant reduction in the prevalence and cumulative incidence of AD at 1 year in probiotic group versus placebo
		Dotterud et al., 2010 [70]	RCT	278 pregnant women	*Lactobacillus rhamnosus* GG (10^10^ colony-forming units), *Lactobacillus acidophilus* La‐5 (10^9^ colony-forming units) and *Bifidobacterium animalis* subsp. *lactis* Bb‐12 (10^10^ colony-forming units), daily from 36 weeks of gestation to 3 months postnatally during breastfeeding (*n* = 138)	Significant reduction in the cumulative incidence of AD in children at 2 years of age versus placebo
		Ou et al., 2012 [71]	RCT	191 pregnant women (with atopic diseases)	*Lactobacillus rhamnosus* GG(ATCC 53103) (1 × 10^10^ colony-forming units), daily from 24 weeks gestation until delivery, and to 6 months to breastfeeding mothers or non-breastfeeding neonates (*n* = 95)	No significant difference in the incidence of moderate to severe AD or plasma IgE in infants at 6, 18 and 36 months
		Cabana et al., 2017 [72]	RCT	184 infants at high risk of AD (mothers with asthma)	*Lactobacillus rhamnosus* GG (10 billion colony-forming units), daily for first 6 months of life (*n* = 92)	No significant difference in the cumulative incidence of AD at 2 years of age
		Boyle et al., 2011 [73]	RCT	250 pregnant women (carrying infants at high risk of allergic disease)	*Lactobacillus rhamnosus* GG (1.8 × 10^10^ colony-forming units/day), from 36 weeks gestation until delivery (*n* = 125)	No difference in the prevalence of AD during the first 12 months of life No significant difference in levels of inflammatory markers (IL-10, IL-13, TNF-α and IFN- γ) in infants whose pregnant mothers received probiotic (n = 31 versus placebo n = 30)
		Allen et al., 2014 [74]	RCT	454 mother-infant pairs	*Lactobacillus salivarius* CUL61, *Lactobacillus paracasei* CUL08, *Bifidobacterium animalis* subsp. *lactis* CUL34 and *Bifidobacterium bifidum* CUL20 (total of 10^10^ organisms/day), maternal supplementation from 36 weeks gestation and infant supplementation to 6 months (n = 220)	No difference in the cumulative frequency of AD at 2 years
		Gore et al., 2012 [75]	RCT	137 infants (3–6 months) with AD	*Lactobacillus paracasei* CNCM I-2116 (10^10^ colony-forming units) (*n* = 45) or *Bifidobacterium lactis* CNCM I3446 (10^10^ colony-forming units) (*n* = 45), daily for 12 weeks	No significant difference in disease severity between groups
		Wu et al., 2012 [76]	RCT	54 children (2–14 years) with AD	*Lactobacillus salivarius* (2 × 10^9^ colony-forming units) plus fructo-oligosaccharide (475 mg) (synbiotic) or fructo-oligosaccharide (475 mg) alone (prebiotic), twice daily for 8 weeks (*n* = 27 in each group)	Significant reduction in disease severity in synbiotic group versus prebiotic group
		Gerasimov et al., 2010 [77]	RCT	90 children (1–3 years) with AD	*Lactobacillus acidophilus* DDS-1, *Bifidobacterium lactis* UABLA-12 (total 5 billion colony-forming units) plus fructo-oligosaccharide (50 mg) (synbiotic), twice daily for 8 weeks (*n* = 43)	Significant reduction in disease severity and use of topical corticosteroids in treatment group versus placebo
		Farid et al., 2011 [78]	RCT	40 infants and children (3 months to 6 years) with AD	*Lactobacillus casei, Lactobacillus rhamnosus, Streptococcus thermophilus, Bifidobacterium breve, Lactobacillus acidophilus, Bifidobacterium infantis, Lactobacillus bulgaricus* (total 1 billion colony-forming units) and fructo-oligosaccharide (synbiotic), twice daily for 8 weeks (*n* = 19)	Significant reduction in disease severity in treatment group versus placebo
		Grüber et al., 2010 [79]	RCT	830 infants with low atopy risk	Mixture of neutral oligosaccharides and pectin-derived acidic oligosaccharides (total 8 g/L) (prebiotics) (*n* = 414), offered up to first year	Significant reduction in AD occurrence up to the first birthday in prebiotic group versus placebo
		Shafiei et al., 2011 [80]	RCT	41 infants (1–36 months) with AD	1 × 10^9^ CFU of seven strain probiotics plus fructo-oligosaccharides (990 mg) (synbiotic),daily for 2 months (*n* = 20)	No significant difference in reduction of disease severity between groups
		Van Der Aa et al., 2010 [81]	RCT	90 infants (<7 months) with AD	*Bifidobacterium breve* M‐16 V 1.3 × 10^9^ colony-forming units/100 mL and a galacto‐/fructo-oligosaccharide mixture (0.8 g/100 mL), (approx. 778 mL daily) for 12 weeks (*n* = 46)	No significant difference in reduction of disease severity between groups
		Drago et al., 2011 [89]	RCT	38 adults (18–46 years) with AD	*Lactobacillus salivarius* LS01 (1 × 10^9^ colony forming units/g), twice daily for 16 weeks (*n* = 19)	Significant improvement in disease severity and quality of life in probiotic group versus placebo
	Acne	Jung et al., 2013 [82]	RCT, open-label	45 females (18–35 years) with acne	Probiotic (*Lactobacillus acidophilus* (5 billion colony-forming units/capsule), *Lactobacillus del-brueckii* subsp. *bulgaricus* (5 billion colony-forming units/capsule), and *Bifidobacterium bifidum* (20 billion colony-forming units/capsule)) or minocycline (standard treatment) or minocycline plus probiotic (probiotic taken twice daily) for 12 weeks (n = 15 in each group)	Significant improvement in disease severity in all treatment groups Significant improvement in disease severity in minocycline plus probiotic group versus other treatment groups
		Fabbrocini et al., 2016 [83]	RCT, pilot study	20 adults with acne	*Lactobacillus rhamnosus* SP1 (3 × 10^9^ colony-forming units/day) for 12 weeks (*n* = 10)	Significant reduction in disease severity in probiotic group versus placebo
		Kim et al., 2010 [85]	RCT	36 males (18–30 years) with acne	Lactoferrin (200 mg) (prebiotic) daily for 12 weeks (*n* = 18)	Significant improvement in disease severity in treatment group versus placebo
	Intestinal borne dermatoses	Manzhalii et al., 2016 [84]	RCT	57 adults (18–42 years) patients with erythematous papular-pustular rash	*Escherichia coli* Nissle 1917 (2.5–25 × 10^9^ colony-forming units/capsule), 2 capsules daily for 1 month (*n* = 37)	Significant amelioration or complete recovery in 89 % of patients with acne, popular pustular rosacea and seborrhoeic dermatitis in probiotic group versus 56 % in placebo
	Plaque psoriasis	Groeger et al., 2013 [87]	Interventional study	26 adults (18–65 years) with plaque psoriasis	*Bifidobacterium infantis* 35264 (1 × 10^10^ colony‐forming units), daily for 8 weeks (all patients received the treatment)	Significant reduction in the plasma concentration of C‐reactive protein and proinflammatory cytokine (TNF‐α) compared to baseline
		Navarro-López et al., 2019 [88]	RCT	90 adults (18–70 years) with plaque psoriasis	1:1:1 of *Bifidobacterium longum* CECT 7347, *Bifidobacterium lactis* CECT 8145 and *Lactobacillus rhamnosus* CECT 8361 (total of 1 × 10^9^ colony-forming units*/*capsule), daily for 12 weeks (*n* = 46)	Significant reduction in disease severity and lower risk of relapse after 6 months in probiotic group versus placebo
	Hand dermatitis	Gulliver et al., 2018 [90]	Open-label	30 adults (over 18 years) with hand dermatitis	*Lactobacillus acidophilus* CL1285, *Lactobacillus casei* LBC80R and *Lactobacillus rhamnosus* CLR2 (30 billion colony-forming units/capsule), daily for 12 weeks (all patients received the treatment)	Significant improvement in 27% of subjects 23% of subjects achieved clear or almost clear hands by 12 weeks Pruritus was improved with 59% of symptomatic patients within 2 weeks
	Dandruff	Reygagne et al., 2017 [91]	RCT	60 males (18–60 years) with dandruff	*Lactobacillus paracasei* NCC 2461 ST11 (1 × 10^9^ colony-forming units), daily for 56 days (*n* = 30)	Significant clinical improvement in probiotic group versus placebo
Topical	Atopic Dermatitis	Blanchet-Réthoré et al., 2017 [92]	Open-label	31 adults (18–75 years) with AD	Heat-treated *Lactobacillus johnsonii* NCC 533 (3.1 × 10^11^ colony-forming units/g), twice daily for 3 weeks (all patients received the treatment)	Significant reduction in *Staphylococcus aureus* load and disease severity in treated versus untreated lesions
		Myles et al., 2018 [93]	Open-label phase I/II trial	10 adults and 5 children (9–14 years) with AD	*Roseomonas mucosa* (dose escalation from 10^3^–105 colony-forming units), adults = twice weekly for 6 weeks, children = twice weekly for 12 weeks (all patients received the treatment)	Significant reduction in disease severity and *Staphylococcus aureus* load compared to baseline measurements
		Butler et al., 2020 [94]	RCT, proof-of-concept	34 adults (18–70 years) with AD	*Lactobacillus reuteri* DSM 17938 (minimum 1 × 10^8^ colony-forming units/g), twice daily for 8 weeks (*n* = 17)	Significant improvement in disease severity after 4 and 8 weeks of use as compared to baseline measurements
		Nakatsuji et al., 2017 [95]	Interventional study	5 adults with AD	Antimicrobial Coagulase-Negative Staphylococci (*Staphylococcus hominis* and *Staphylococcus epidermidis* (1 × 10^5^ colony-forming units/cm^2^)) isolated from human skin, single dose on one forearm (vehicle control on other forearm)	Significant reduction in Staphylococcus aureus burden on treatment forearm versus vehicle control forearm
	Acne	AOBiome Therapeutics, 2017 [96]	RCT, Phase 2b	358 adults with acne	*Nitrosomonas eutropha* (dose not specified), twice daily for 12 weeks	Significant reduction in disease severity in probiotic group versus vehicle control
		Muizzuddin et al., 2012 [97]	Interventional study	29 females (25–55 years) with acne	*Lactobacillus plantarum* (at 1% or 5% concentration) or Triclosan (an antibacterial agent) (at 0.1%), twice daily for 2 months (groups of 9–10 each)	Significant reduction in disease severity in probiotic (5 % concentration) group as compared to baseline measurements
		Bateni et al., 2013 [98]	RCT	26 females (18–39 years) with acne	Glucomannan hydrolysates (prebiotic) (5% (w/v)) or standard treatment (antibiotics), twice daily for about 6 weeks	Significant improvement in disease severity at 20 and 40 days for both treatments
	Reactive skin	Guéniche et al., 2010 [99]	RCT	66 females with reactive skin	*Bifidobacterium longum re*uter lysate (5 %), twice daily for 2 months (*n* = 33)	Significant decrease in skin sensitivity and increased skin resistance to physical and chemical aggression in probiotic group versus placebo

Significant findings are reported at *p* ≤ 0.05.

*RCT* randomized controlled trial, *AD* atopic dermatitis.

The potential for probiotics to treat paediatric AD has been a focal point of research. A number of randomized controlled trials (RCTs) have reported a reduction in disease severity and inflammatory markers following administration of oral probiotics to children with AD [59–67]. Certain large studies have observed significant preventative effects of oral probiotic supplementations for paediatric AD, when given both pre- and postnatally to mothers and their infants [68–70]. Conversely, other trials have noted no differences between oral probiotics and placebo in preventing or improving the clinical presentation or inflammatory markers seen in children with AD [71–75]. Several studies have assessed the combination of probiotics and prebiotics (synbiotics) in this AD population and reported decreases in disease severity [76–78]. Interestingly, studies using prebiotics alone had some activity against AD [76, 79]. However, other trials have reported the significant lack of synbiotic activity in this population [80, 81].

The use of oral probiotic interventions in adult acne has similarly gained significant attention. Trials have demonstrated clinical improvement using probiotics alone and in combination with standard treatment [82–84]. Nevertheless, one trial reported that probiotics alone provided little benefit, yet reported significant clinical improvement when combined with the potential prebiotic lactoferrin [85, 86]. Other recent studies have shown potentially beneficial effects of oral probiotics in plaque psoriasis [87, 88], adult AD [89], hand dermatitis [90], papulopustular rosacea, seborrhoeic dermatitis [86] and dandruff [91].

Although a number of studies have investigated the potential of oral probiotics, trials assessing the effect of topical probiotic and prebiotic interventions in skin disease are limited. Reduced AD disease severity in both adults and children treated with topical probiotics has been reported [92–94]. Further, interventional studies have observed reduced *S. aureus* colonization and one study even reported decreased *S. aureus* burden in patients after just a single application of a lotion containing antimicrobials isolated from human skin commensals [95]. In adult acne, a phase 2 clinical trial reported a significant reduction of acne severity and inflammatory lesions with the use of a topical probiotic spray, although this study seems yet to be published in full [96]. Individuals treated with a bacterial strain in an oil-in-water formulation displayed reductions in erythema and acne lesion size, along with reparation of the skin barrier [97]; another study reported improvement in skin health associated with a topical probiotic spray used in acne patients [98]. In addition, one RCT demonstrated that a single strain probiotic cream significantly reduced skin sensitivity and increased resistance to chemical and physical injury in females with reactive skin [99].

While some evidence exists to support the use of certain probiotic and/or prebiotic therapies in skin disease, the heterogeneity of outcomes between studies is a major limitation. This may be due to variations in treatment regimens, participant demographics, inclusion and exclusion criterion and the use of single versus multi-strain preparations, among others. Indeed, one study demonstrated the importance of strain choice on affecting the prevalence of AD [66]. Moreover, many studies are underpowered and limited by their small sample sizes and short follow-up. The transient nature of many skin diseases may necessitate longer studies to see true results. Only a few studies reported the ethnic background or skin type of participants, and even fewer commented on diet, even though host microbiome diversity associated with cultural and genetic factors is well appreciated [100]. Currently active and recruiting trials include investigations of oral probiotic supplementations in paediatric and adult AD, acne, as well as topical probiotics in paediatric AD [101]. These studies and others will hopefully help to further clarify the usefulness of these interventions in skin disease, where microbial dysbiosis is a well-established pathogenic feature.

## Conclusions

Significant advances have been made during recent years in describing the composition of the microbiome on the skin and the changes in bacterial communities that associate with, or sometimes precede, skin inflammatory disorders such as AD. However, substantial gaps in our knowledge on the microbiome still exist. In particular, the functional basis for microbe-host communication within the skin is still poorly described. In addition, novel probiotics and not just the traditional probiotic strains need to be clinically tested. Furthermore, microbial components or their metabolites should also be examined, in particular the application of these novel microbial drugs to the diseased site must be better explored.
